# An Epochal Moment? The COVID-19 Pandemic and China’s International Order Building

**DOI:** 10.1177/0043820020945395

**Published:** 2020-09

**Authors:** Nicholas Ross Smith, Tracey Fallon

**Affiliations:** University of Nottingham Ningbo China

**Keywords:** Pandemic, China’s Rise, Coronavirus, COVID-19, International Order, International Friendship, International Relations, Global Leadership Void, World Order, Health Crisis, International Politics, Global Crisis Response, Humanitarian Aid

## Abstract

This article considers the potential for the COVID-19 pandemic to represent an epochal moment in international politics, one which China can use as a window to maximize its international order-building efforts. It is argued that China’s putative international order-building efforts to date have faltered, in part, due to an inability to create meaningful friendships with prominent international powers. However, the COVID-19 pandemic allows China to play a key humanitarian role in heavily afflicted countries and, through this, perhaps an opportunity to forge more meaningful friendships. It is argued that China is trying to attach its concept of friendship to its humanitarian assistance while also stepping into the clear international leadership void that exists at the moment. This is not without faults or missteps, but the lasting impact could be significant for international politics and the global order.

## ¿Un momento epocal? La pandemia de covid-19 y la construccion del orden internacional por parte de China

Este artículo considera el potencial de la pandemia de COVID-19 para representar un momento de época en la política internacional, uno que China puede usar como una ventana para maximizar sus esfuerzos de construcción del orden internacional. Se argumenta que los supuestos esfuerzos de construcción del orden internacional por parte de China hasta la fecha han fallado, en parte, debido a su incapacidad de crear amistades significativas con potencias internacionales prominentes. Sin embargo, la pandemia de COVID-19 le ha permitido a China jugar un papel humanitario clave en países muy afectados y, a través de esto, tal vez una oportunidad para forjar amistades más significativas. Se argumenta que China está tratando de vincular su concepto de amistad a su asistencia humanitaria al mismo tiempo que aprovecha en el claro vacío de liderazgo internacional que existe en este momento. Esto no está exento de fallas o errores, pero el impacto duradero podría ser significativo para la política y el orden internacionales.

## 一个划时代的时刻？ COVID-19 大流行与中国的国际秩序建

本文研究了COVID-19大流行代表国际政治中一个划时代时刻的可能性, 中国能将这一时刻作为机会之窗, 将其为国际秩序建立所作的努力最大化。论证认为, 中国公认的国际秩序建立工作至今出现衰落, 这部分归因于其无法与重要的国际强权建立有意义的友谊关系。然而, COVID-19大流行让中国能在受严重影响的国家中发挥关键的人道主义作用, 并借此可能有机会与其形成有意义的友谊关系。论证认为, 中国正努力将其友谊概念与其人道主义援助相联系, 同时走进当前清晰的国际领导者真空。此举并非没有缺点或失误, 但其产生的长期影响对国际政治及秩序而言可能是显著的。关键词

**T**he impact of COVID-19 has been so rapid and globally enveloping that it deserves to be considered as a potential epochal moment for international politics. Since emerging in Wuhan, China, the virus has swept across the globe and has not only caused significant public health costs, especially in Asia, Europe, and North America, but also crippled markets, closed borders, and resulted in tit-for-tat finger-pointing between China and the United States. Although there is much uncertainty to how the COVID-19 pandemic will develop in the coming months and years, it is likely to cause a lasting impact that will be felt in international politics, particularly with regard to international power and order.

Epochal moments in international politics are critical junctures or tipping points that disrupt the *status quo*, resulting in new realities once the dust settles. They are also crucial for order in international politics. While international systems and orders are hardly static and are always evolving, epochal moments make monumental changes suddenly. Think of historical moments like the Thirty Years War or the defeat of Napoleon. These events were critical junctures of great magnitude for European politics at the time. The Thirty Years War ushered in a new system in Europe based on the concept of sovereignty while the defeat of Napoleon led to the Concert of Europe, a system in Europe based on the concept of balance of power (Gross 1948).

Epochal moments have also been instrumental to the trajectory of the U.S.-led international order, which had its origins in the aftermath of World War II (WW2). WW2 was arguably the most significant critical juncture experienced in this so-called Westphalian era, leading to significant material and ideational power shifts (Smith 2020). The United States emerged out of WW2 as the world’s strongest power—albeit closely followed by the Soviet Union—and went about crafting an international order while the Soviet Union offered an alternative order (Mearsheimer 2019). The collapse of the Soviet Union in 1991 was a further epochal moment, resulting in another significant material and ideational power shift. The result was the entrenchment of the United States as a global hegemon presiding over an unrivaled (liberal) international order (Ikenberry 1999).

In the years since the United States’ hegemonic moment in 1991, the international order it presides over has come under threat. The cracking of this international order has been, in part, self-inflicted as the United States engaged in disastrous wars in Afghanistan and Iraq as well as numerous other foreign policy blunders (Duncombe and Dunne 2018). More recently, the election of President Donald Trump dealt a further blow, leading Barry Posen (2018, 20–21) to argue that the U.S. grand strategy has switched from “liberal” to “illiberal” hegemony: a strategy whereby the United States retains its “superior economic and military capability and role as security arbiter for most regions of the world, but he has chosen to forgo the export of democracy and abstain from many multilateral trade agreements.”

But the cracking of the U.S.-led international order has also been aided by the emergence of challengers to the U.S. hegemonic power position, none more so than China. China’s rise over the past two decades has been remarkable (Brooks and Wohlforth 2016). Its economy is now the world’s largest and its military capabilities have also grown, albeit not to the point of challenging the United States (Champion and Leung 2018). China also weathered the global financial crisis better than the West and, although it maintains something of a peaceful rise narrative, has sought to exert much more influence, at first regionally in Asia—such as through the Belt and Road Initiative (BRI) and the Asian Infrastructure Investment Bank (AIIB) (Liu 2020)—but also increasingly on a global scale (Zhang 2016). China’s rise has led it to covet a fairer international order, one which privileges the United States less and recognizes China’s rise (Breslin 2013). Indeed, as U.S. willingness to lead eroded further in Donald Trump’s first term as president, Xi Jinping (2017) sought to insert China into the void, famously stating at the 2017 World Economic Forum in Davos that China has

blazed a development path that suits China’s actual conditions. This is a path based on China’s realities . . . by drawing on both the wisdom of its civilization and the practices of other countries in both East and West. In exploring this path, China refuses to stay insensitive to the changing times or to blindly follow in others’ footsteps . . . We are not jealous of others’ success; and we will not complain about others who have benefited so much from the great opportunities presented by China’s development. We will open our arms to the people of other countries and welcome them aboard the express train of China’s development.

This article examines whether COVID-19 can be an epochal moment that China can utilize to aid its putative international order building. To do this, first the importance of meaningful friendships to international order building and China’s struggles in cultivating such friendships are examined. Second, the material and ideational costs of the COVID-19 pandemic are evaluated while China’s emerging relative advantage over the rest of the world is gauged. Third, China’s efforts to combine its humanitarian assistance with friendship cultivation is assessed, with a focus on Italy. Last, it is concluded that COVID-19 does represent a useful window that China can use to foster real international momentum with regard to its international friendship and order building.

## The Importance of Meaningful Friendships

Despite plenty of investment, enthusiasm, and ambition, China’s international order building has yet to produce any tangible results to date. One hurdle it has encountered has been an inability to cultivate meaningful friendships with other countries, especially powerful ones. Friendship has long been crucial to international order building. As Roshchin (2006, 602) argues, at the time of the Treaty of Westphalia, after the “dissolution of the *Respublica Christiana*, abandonment of Canon Law together with Papal authority, and the spread of the Reformation,” friendship “became one of the few concepts mediating the emergence of the modern political order.” Therefore, given that international politics still exists within something of a Westphalian system, friendships have played an incredibly important role in the rise and fall of different orders in the 400-odd years since.

It is not for lack of effort as China has long realized that friendships are crucial to order building, and it has attempted to cultivate friendships with many different countries across the globe (Strüver 2014). One example of this is their concept of “all-weather friend,” which is used by China to describe a relationship in which there is mutual trust and firm political support (Li and Chang 2015). To date, 14 countries have been called China’s “all-weather friend”; in Asia: Pakistan (by Xi in 2015) and Yemen (by Hu Jintao in 2006); in Africa: Zimbabwe (by Xi in 2015), Ethiopia (by Xi 2013), Kenya (by Xi 2013), Tanzania (by Xi in 2013), Mali (by Hu in 2010), Namibia (by Yang Jiechi in 2010), Egypt (by Zhu Rongji in 2002), and Zambia (by Mao in 1967); in Europe: Malta (by Li Keqiang 2014), Serbia (by Xi in 2011), and Romania (by Xi in 2009); and in South America: Brazil (by Hu in 2004) (Li and Chang 2015).

Two problems emerge when canvassing China’s list of “all-weather friends” with regard to order building. First, none of these countries, outside of arguably Brazil, are prominent international agenda setters. China’s desire for parity in the international system requires that more prominent states buy into their international vision; otherwise, their order building will fail to challenge the existing order(s). Second, most of these friendships are unequal and are based not on mutual affinities but rather China’s use of largesse, especially the so-called “no strings attached” loans (Gonzalez-Vicente 2015). States sign up to be “friends” with China to reap the benefits on offer rather than agreeing with their vision of the international order. Thus, these friendships lack the substance needed for order building.

Russia, however, looms large as one prominent international power that China has seemingly become friendly with. Under the leaderships of Xi Jinping and Vladimir Putin, respectively, China and Russia have taken noticeable steps to solidify their relationship. The state of affairs appears so rosy that the term “bromance” is now widely used to characterize this blossoming friendship between the two (Kempe 2019). And beyond the obvious hyperbole of there being a bromance of sorts, there is some concrete evidence to suggest a strong connection between the two leaders exists. Since Xi came to power in 2013, there have been more than 25 meetings between the two, the greatest interaction with another world leader for each (Zhou 2019). Beyond the seemingly close personal relationship between Xi and Putin, the two countries have experienced some notable alignment with regard to traditional security engagement. Not only do both regularly interact through the Shanghai Cooperation Organization (SCO), they have also institutionalized, since 2015, the “China-Russia Northeast Asia Security Dialogue” as well as undertaking numerous joint military exercises (Korolev 2019).

However, while Sino-Russian cooperation is strong with respect to security matters, it is still relatively weak in terms of trade relations: Russia has significantly higher two-way trade levels with the European Union (EU), while China’s two-way trade with Russia does not even register on its top 10 list. Furthermore, there is a potential for Russia to become nervous about Chinese encroachment into its “sphere of privileged interest,” especially as China’s flagship policy, the BRI targets erstwhile Soviet Central Asia (Samokhvalov 2018). In addition, although both ostensibly believe in respect for sovereignty (as well as mutual displeasure with the liberal international order), there is not much else (ideationally) binding the two together and there is a significant history of mistrust (and some conflict) between the two also (Bekkevold and Lo 2018).

As the Russian example shows, China has generated friendships that appeal to strategic or normative affinities, but deeper and lasting friendships usually need stronger ideational glue as well. To this end, cultural diplomacy is one area in which China has invested heavily in recent times to try and cultivate more meaningful friendships. Initiatives under this umbrella included proliferating Confucius Institutes (CI) and Confucius Classrooms abroad and attracting international students to study in China (Hartig 2015). Piggybacking on the attraction of China’s cultural resources and employment desires for students attracted by the “rise of China,” China has utilized Mandarin education as a way of cultivating future friends for China who “know China and are friendly to China” (“zhi Hua you Hua”) and carry across its visions of an international order (Fallon 2014). Xi Jinping, when vice-president, told international students from ASEAN countries that “I hope that everyone after their studies will become friendly emissaries who know and are friendly with China, building communication bridges between China and each ASEAN country, and be forever a friend of China, China’s best friend” (*China News* 2010).

Despite increased efforts to cultivate meaningful friendships, one of the greatest barriers to this involves China’s national identity narratives. Chinese national identity, like other national identities, is predicated on a distinction with “Others.” Looming large among its significant “Others,” are Japan and the United States (Gries 2004). Although Japan has traditionally played the most conspicuous role as the “Other,” in recent years, the United States has eclipsed Japan’s role as the main identity reference point (Atanassova-Cornelis 2012). According to Chinese nationalistic discourses, the United States has victimized China and held it back from playing a more prominent international role. National narratives of a rising China following a century of national humiliation at the hands of foreign powers have resonated strongly with the general public since the reintroduction into the education system post-1989 (Callahan 2010). These narratives of humiliation now work to deflect dissension from the state to the enemy outside. Thus, a lack of friendships with particular powers fuels China’s domestic identity narratives and state legitimacy. Yet these very narratives present a barrier to the development of close friendships for their negative view of international “Others.”

When China is misunderstood, it not only reinforces China’s positive distinction with the negative “foreigner,” but can at times provoke anxiety-fueled reactions that lead to declarations from the Chinese state and high-profile figures that the transgressors have “hurt the feeling of the Chinese people” (Barmé 2011). While friendship should offer a way of calming anxiety through recognition of the national narratives from international others, misrecognition of those narratives are anxiety generators (Berenskoetter 2007). For example, writing about the 1989 Tiananmen incident, meeting with the Dali Lama, border disputes, or sovereignty issues will gather the condemnation of “hurting the feelings of the Chinese people” (Bandurski 2016). This has provoked an “us-against-the-world” attitude among many in China (Wang 2012). This and the state’s patriotic education campaign instituted in schools from that time produced a strong defensive type of nationalism that filters through into its foreign policy making.

On the flip side, while China has continued to struggle to create meaningful lasting friendships, the United States, despite all its apparent self-inflicted wounds, still maintains important friendships with numerous key partners. Undeniably, the U.S.-led international order is underpinned and solidified by many key international friendships between the United States and its partners. The most important has been the transatlantic friendship between the United States and the United Kingdom. This is a relationship not built solely on strategic interests but also on a deep mutual normative and cultural understanding as well as being rooted in numerous historical events. This friendship has shaped international politics inexorably over the years, from cooperation in the two world wars of the 20th century to mutual assistance in building the post-1945 liberal international order, to an ongoing partnership in the post-Cold War era (Marsh and Baylis 2006).

Of course, the relationship has not been without its tensions. Particularly problematic was the changing power relation between the two, especially as the United States rose to become the unquestioned hegemon in international politics post-1945, while the United Kingdom declined to a point of clear subordination (Dobson 1995). However, it was arguably the deep friendship these countries created that enabled them to weather these issues and maintain a robust, friendly relationship to the present day, even amidst the chaos of Brexit and Trump (Michaels 2019). Along with the United Kingdom, the United States also has deep friendships with numerous other important players such as the EU and also, individually, with many EU member states such as Denmark, Germany, Ireland, Italy, Poland, Portugal, and the Netherlands (Shapiro and Pardijs 2017). Outside of Europe, the United States also has important friendships which cover the entire globe, including Canada, Australia, Japan, Saudi Arabia, and the Republic of Korea (Katz and Quealy 2017).

However, COVID-19 is a massive test for the United States and whether it can come through the crisis with its international reputation and its international friendships intact remains to be seen. Given that it was already losing its appetite to underwrite the international order’s provision of public goods—such as international security, international trade, international capital flows, and freedom of navigation—shirking a leadership role during the COVID-19 pandemic could leave the door open for new leaders, and perhaps, new orders (Acharya 2017).

## COVID-19 as a Window of Opportunity for China

The COVID-19 pandemic has been particularly bad for China domestically. Given that the pandemic first emerged in the Chinese city of Wuhan, much of the initial damage of the pandemic was in China. China was forced to close Hubei province and quarantine the major settlements there, including Wuhan, a city of roughly nine million people. Soon after, much of the country was quarantined and citizens were forced to stay indoors to contain the spread of the virus. The health costs of the pandemic are already significant—including more than 84,000 cases and 4,500 dead at the time of writing (World Health Organization [WHO] 2020). Initially, it appeared to some observers that the political costs for the Chinese government would be large, particularly the brewing outrage over the death of the whistle-blower doctor, Li Wenliang, and the revelation of the initial mishandling of the situation (Anderlini 2020).

However, the Chinese government recovered quickly by co-opting Dr. Li’s “martyrdom” from the outpouring of calls for greater transparency and freedom of speech to enshrining him and other medical staff who died within the national rankings of “martyr” (Su 2020). There has also been an embrace of the slogan: “Come on Wuhan, Come on China” (“Wuhan Jiayou, Zhongguo Jiaoyou”; Congyou 2020). Together, this has worked to bolster support for state-led measures to combat COVID-19, allowing individuals to participate in the “we-ness” of a national effort. Thus, rather than legitimacy, the greatest political challenge will be the economic costs of the outbreak in China, particularly as many of China’s strongest trade partners are all suffering from outbreaks now as well. The World Bank’s most recent forecast suggested China could experience zero growth in 2020, which would be the first time to drop below 3.9 percent since 1976 (He 2020).

Internationally, the COVID-19 outbreak came on the back of a problematic year for China’s international image in 2019, and by extension, its cultivation of friendships. Further revelations about Xinjiang were printed in major international newspapers, the Hong Kong protests spiraled out of control, the fiasco with the NBA and other Western companies exposed China’s naked use of its market power, and backlashes against its flagship BRI policy occurred in numerous countries, many of which were expected to be key partners for China’s envisaged new world order (Smith and Fallon 2019). If 2020 was expected to be a year China could reset its international image, the initial COVID-19 outbreak further damaged China’s international image. The prevalence of the wild animal trade, viral videos of people being sealed into their apartments or forcibly removed to quarantine, and doubts over COVID-19 deaths, provided further evidence of China’s growing totalitarianism in front of a rapt international audience (Lei and Qiu 2020).

But the rapid transformation of COVID-19 from an isolated epidemic in Hubei province into an international pandemic, one which few Western countries seem capable of containing, is a potential game changer for China. Although China’s initial handling of the outbreak was heavily criticized, the fact that China stemmed the tide against COVID-19 in less than two months now looks quite incredible *vis-à-vis* those countries most severely hit in Europe and North America. Given China has more than 100 cities with at least one million people (*The Guardian* 2017), the fact they have managed to keep deaths per million citizens down to three to date is impressive (WHO 2020). Part of this success has been their ability to repurpose the grid management system generally used in Xinjiang and Tibet as a means of social control, to now ensure a highly efficient lockdown (Pan, Yao, and Zhao 2020). Comparatively, many Western countries have been unable (or unwilling) to employ as rapid or as authoritarian measures and have suffered significantly higher COVID-19 deaths per million citizens. At the time of writing, Belgium led the way at more than 800, followed by the United Kingdom (610 per million), Spain (580), and Italy (560), while those of the United States continue to rise (350) (WHO 2020). In fact, no seriously afflicted Western country has been able to keep the death rate as low as the published Chinese figures.

China’s rapid recovery seemingly gives it an advantage over the rest. Of course, as already mentioned, the internal costs are going to be significant and how the political and economic challenge will play out for the Chinese Communist Party (CCP) in the coming year or two is another question. But the fact that China appears to have the virus under control and has implemented an effective system of surveillance and control that few other countries have the resources or legal wiggle room to copy, it seems China could end up benefiting from being able to get its country back up and running before the rest. Already in early March, less than two months after shutting down, China took steps to kick-start its economy again, working with businesses and employers on plans to increase productivity and children returning to schools in less affected areas (McMorrow 2020).

The potential swings are not just material but ideational, because the COVID-19 pandemic is also potentially a *tabula rasa* for China’s international image and its efforts to cultivate friendships. Even though the Chinese model for combatting the outbreak of COVID-19 is not easy or realistic for most countries to follow, China is still a source of important information and expertise given it has been first in dealing with the outbreak. As many European countries have struggled to deal with the spread, there have been significant calls from the intelligentsia in these countries to take seriously China’s experience and to find ways to use this to drive policy choices (Buell 2020). China has also been actively sharing its experience in medical, customs, and quarantine management with officials and experts around the world through video conferencing (Shuang 2020a). The state has effectively shifted the discourse from a struggling China (“Come on China” [“*Zhongguo jia you*”]) to that of responsible supporter (“China supports”[“*Zhongguo zhiyuan*”]).

Another area of both material but also ideational advantage is China’s increasing position as the world’s largest producer of medical supplies. Although China’s production of high-tech medical equipment is much smaller than countries like Germany and the United States, when it comes to medical supplies it is the undeniable market leader, providing 43 percent of global imports of protective medical equipment (Brown 2020). Indeed, combatting the COVID-19 outbreak requires significant quantities of testing swabs, protective masks, surgical gowns, and hand sanitizer, of which China is the world’s leading producer. Since the outbreak, and despite the internal shutdown, China has upped its production of the N95 mask, producing 116 million masks per day which is roughly 12 times what it was producing before (Ren 2020). The fact that China has the capacity, even under lockdown, to keep producing the medical supplies needed to successfully combat COVID-19 means it is well placed to play the role of a humanitarian power by offering access to these supplies. Indeed, on that front, after China’s internal situation began to stabilize in mid-February, it started providing humanitarian assistance to heavily afflicted countries. Even Jack Ma, the famed Chinese tech billionaire, has been personally providing face masks and equipment to heavily afflicted countries, including the United States (Ng 2020). China’s assistance has been termed “mask diplomacy” and, according to B. Wong (2020), it symbolizes China’s efforts to “rise to the challenges of global leadership” and “provide relief to siblings and friends.”

## COVID-19 and the Forging of Meaningful Friendships for China

Europe offers an interesting case study to evaluate whether the onset of the COVID-19 pandemic has offered China a chance to make some ground there with regard to its friendship initiatives. At a continental level, Europe quickly surpassed Asia in terms of total COVID-19 cases and deaths. And, unlike China, the governments there have been far less effective at stopping the spread of the virus, meaning a much more protracted outbreak has occurred which will likely result in larger societal and economic costs. Italy, for example, reported its first case on January 29, 2020, but did not reach its peak in terms of new cases per day until March 21, 2020, and it was not until mid-May that a significant drop occurred (WHO 2020). Comparatively, China’s new cases graph was much steeper—at its peak, 15,000 new cases occurred in one day—but dropped much more quickly once China’s strict measures were implemented (WHO 2020). It took China roughly three weeks to get the virus under control, while Italy took more than two months before flattening the curve significantly from its peak (WHO 2020).

Once it became apparent that Italy was facing a significant outbreak of COVID-19, its leaders initially appealed to the EU, and the member states of the EU individually, for help. However, the EU proved ineffectual in providing an EU-wide solution and also proved powerless to stop Germany and France imposing limits on the export of protective medical equipment (Braw 2020). This elicited significant outrage in Italy, a country which already had significant anti-EU sentiments among large swathes of its citizenry (Fortuna 2020). The United States, also, initially failed to help Italy, or Europe more broadly, only providing some assistance later on while remaining largely muted (Erlanger 2020).

The perceived lack of solidarity and urgency shown by the EU and the United States in Italy created an initial vacuum that the Chinese stepped into. On March 13, 2020, China began by sending nine experts involved in the Wuhan response to Italy to offer advice. This was accompanied by the Chinese Red Cross providing 31 tons of medical equipment—including high-tech equipment like respiratory devices, electrocardiographs, and ventilators (B. Wong 2020). On March 17, Xi Jinping stated that China would continue to send more medical experts to Italy to help with the worsening conditions there, and by March 25, China had sent three teams. Thus, while Italian officials have openly criticized its European friends, at the same time, they gave thanks for China’s responses (Barigazzi 2020).

In the past, scholars have critiqued China’s attempts at public diplomacy, noting its weakness at involving non-state actors and prioritizing top-down, state-lead initiatives which end up producing sharp, not soft, power (see, for example, Walker 2018). But now, China’s publicizing of its generosity has seemingly taken a page from a textbook of public diplomacy, and looks to Chinese non-state actors’ donations in the form of tech giants Huawei and Xiaomi, as well as and retailers such as Jack Ma’s Alibaba and Richard Liu’s JD.com (Ireland 2020). Less obvious state actors such as the Bank of China, and sub-national state local government donations, plus sharing expertise of paired sister cities have also been involved. Alibaba’s sharing of AI technology tools for detecting COVID-19 infections in computed tomography (CT) scans to European hospitals provides high-tech solutions with seemingly no commercial strings attached (Cogley 2020). This kind of Chinese support not only appears to show leadership at the top but a multi-pronged approach that highlights the aid and donations from multiple Chinese actors, and as such has the comprehensive potential to improve the overall national image of China.

Attached to China’s recent global show of largesse lies significant referencing to friendship. From early March when the tide began to turn with China’s controls and increasing cases outside, the CCP’s framing of China’s assistance communicated through the Ministry of Foreign Affairs (MFA) briefings, centered on reciprocity of friendship and in the interconnectedness of existence. The repeated use of the phrase “shared future of mankind” has become commonplace in official press conferences of the Chinese MFA, positioning China as a humanitarian power (*CGTN* 2020). But China has also signaled its self-identity in its approach through reference to traditional sayings, such as “you throw a peach to me, and I give you a white jade of friendship” (Shuang 2020b). Such signals indicate China’s donations as a reciprocal aspect of its friendship.

This broad signaling of friendship is also supplemented with more overt references for the countries China has been working closely with since the spread of the virus. For Italy, the support is framed on the interconnectedness of humanity. One example is the use of the quote “we are waves of the same sea” from the Roman stoic philosopher Seneca on the donation boxes from Chinese high-tech firm, Xiaomi. In addition, the Chinese Embassy in Rome has tried to highlight the previous strong ties between the two countries, especially after Italy helped China after the Wenchuan earthquake in May 2008. The Chinese Embassy stated that “Maybe you have forgotten about it, but we will always remember. Now it’s our turn to help you” (Tzogopoulos 2020). As the situation has worsened in Europe, China has made similar “friendly” overtures to Spain, France, and Germany, among others (Lau 2020a). Brzezinski argues that China’s “aid blitz is also intended to position China as the great power that came to the assistance of Europe” (*Atlantic Council* 2020). It is the reiteration of friendship between China and these individual countries that is the key concept gluing this “aid blitz” together.

Outside Western Europe, China has been even more explicit in signaling to its so-called “all-weather friends.” In response to Pakistan’s initial assistance, an official Chinese spokesperson stated that

Right after the epidemic broke out, Pakistan mobilized the whole nation to provide assistance to China, which we will never forget . . . Reciprocating an act of kindness is our nation’s fine tradition. Now the situation in China is getting better and the number of confirmed cases in Pakistan has increased. The Chinese leadership has made it clear that we will do all we can to support our Pakistani friends. (Shuang 2020b)

Rhetorical responses to Africa were similar: “China and Africa are good friends, partners and brothers. We have long been supporting and helping each other” (Shuang 2020c). And in providing aid to Serbia, Xi Jinping stated, “China and Serbia are comprehensive strategic partners . . . the hard-as-iron friendship of the two countries, and of the two peoples, shall last forever” (Lau 2020a).

This emphasis on reciprocity casts China as regarding its friends kindly. It also underlines a sharp contrast with the “unfriendly” activities of the United States since the outbreak went global. In early February when the outbreak was mostly only seriously affecting China, the Chinese state condemned the U.S. travel warnings to China, pointing out how that, and associated public comments, lacked the friendly support offered by other parts of the international community. This was highlighted as revealing the lack of friendship emanating from the United States through the evocation of the phrase: “a friend in need is a friend indeed” (*China Daily* 2020). In addition, Xi Jinping, in a conference call with G20 leaders iterated the need for “stronger international cooperation” while remarking, “At the most difficult moments for China, many members of the international community provided China with sincere help and support. We would remember and value this friendship forever” (Lau and Churchull 2020).

However, China’s efforts have not been without issues. One major credibility hit has been problems identified with the quality of supplies purchased from China (*BBC News* 2020). Accusations have also surfaced that purported “donations” were actually purchases of shipments (Ferraresi 2020). And the war of words over the suspected origins of the outbreak, while casting clouds of doubt and misdirection over what is known about the virus, serves as an ongoing reminder to global publics that, despite China’s recent efforts in providing humanitarian assistance, the first known outbreak of COVID-19 was in China (Cole 2020). Last, while the donations from Chinese companies—such as Jack Ma’s donation of ventilators to New York—have been well received and openly thanked, the Chinese state’s attempts to concurrently use the donations to improve its national image (or to bully afflicted countries) have not gone unnoticed. This has led foreign publics to question the sincerity of the Chinese offers of friendship (see, for example, E. Wong and Mozur 2020).

## Conclusion: Can the United States Stave off China’s International COVID-19 Gains, or Are We Entering a New Epoch?

China’s noticeable efforts to use the COVID-19 pandemic as a springboard for cultivating deeper friendships to help foster its visions for, and potential impact upon, a new international order has not gone unchallenged, especially from the United States. After President Trump initially praised the Chinese efforts in combatting the outbreak in February 2020 (Steinbuch 2020), since March 2020 the United States has sought to push back hard on China’s narrative. The CIA has used its intelligence to challenge the official Chinese statistics, while officials, including Trump, have been calling the virus “China virus” or “Wuhan flu,” to assert blame on China for the global pandemic (Mangan 2020). The EU has also joined the United States by launching a campaign to fight purported disinformation about its lack of intervention or assistance with regard to Italy’s experience, while also pointing out inaccuracies in China’s narrative (Lau 2020b).

Noticeably absent from the U.S. response to China so far has been a convincing assertion of global leadership (Gramer and Lynch 2020). Perhaps what is inhibiting a strong U.S. international presence at the moment is the hit it has taken domestically since COVID-19 first appeared there in mid-February. At the time of writing, the United States is the most seriously afflicted country, having almost three times the total cases (the United States has more than 2 million) as the second most afflicted country, Brazil (WHO 2020). America is suffering from significant shortages in medical supplies and an over-capacitated health system while failing to significantly flatten the curve of total cases and deaths.

Clearly, China is not winning hearts and minds everywhere. Many of its efforts to assist other countries in the pandemic have been undermined by either shoddy products, a failure to deliver on promises, or pressing too hard. Furthermore, ongoing revelations as to China’s initial mishandling of the situation and the result this had in enabling the outbreak to become a pandemic will continue to undermine their claims to global (health) leadership. But for COVID-19 to be something of a window to an epochal moment, China does not need to win everyone over. China’s willingness to help afflicted countries, issues notwithstanding, has reinvigorated its efforts to cultivate friends and, judging by Italy and Spain, it has made significant strides in countries they previously had failed to create *truly* meaningful international friendships with. At the same time, continued inaction by the United States will likely further erode those friendships crucial to maintaining the international order that country formerly presided over. At the very least, COVID-19 is likely to disrupt the *status quo* and usher in a new period, if not epoch, where China and the United States compete to etch out international orders more favorable to their grand strategic aims. Cultivating and maintaining international friendships will remain a central feature of this struggle.
